# Cytokine expression profiles of immune imbalance in post-mononucleosis chronic fatigue

**DOI:** 10.1186/1479-5876-10-191

**Published:** 2012-09-13

**Authors:** Gordon Broderick, Ben Z Katz, Henrique Fernandes, Mary Ann Fletcher, Nancy Klimas, Frederick A Smith, Maurice RG O’Gorman, Suzanne D Vernon, Renee Taylor

**Affiliations:** 1 Division of Pulmonary Medicine, Department of Medicine, University of Alberta, WMC 2E4.41 WC Mackenzie Health Sciences Centre, 8440 112 Street, Edmonton, AB, T6G 2R7, Canada; 2 Division of Infectious Diseases, Anne and Robert H. Lurie Children’s Hospital of Chicago, Chicago, IL, USA; 3 Department of Medicine, University of Miami, Miami, FL, USA; 4 Miami Veterans Affairs Medical Center, Miami, FL, USA; 5 Department of Pathology, Feinberg School of Medicine, Northwestern, Chicago, IL, USA; 6 CFIDS Association of America, Charlotte, NC, USA; 7 Department of Occupational Therapy, University of Illinois at Chicago, Chicago, IL, USA

**Keywords:** Cytokines, Infectious mononucleosis, Chronic fatigue, Classification model

## Abstract

**Background:**

As Chronic Fatigue Syndrome (CFS) has been known to follow Epstein-Bar virus (EBV) and other systemic infections; our objective was to describe differences in immune activation in post-infective CFS (PI-CFS) patients and recovered controls. We studied 301 adolescents prospectively over 24 months following the diagnosis of monospot-positive infectious mononucleosis (IM). We found an incidence of CFS at 6, 12 and 24 months of 13%, 7% and 4% respectively.

**Methods:**

Using chemiluminescent imaging we measured the concentrations of IL-1a, 1b, 2, 4, 5, 6, 8, 10, 12 (p70), 13, 15, 17 and 23, IFN-γ, TNF-α and TNF-β in duplicate plasma samples available in bio-bank from 9 PI-CFS subjects and 12 recovered controls at 24 months post-infection.

**Results:**

Standard comparative analysis indicated significant differences in IL-8 and 23 across subject groups. In constructing a linear classification model IL-6, 8 and 23 were selected by two different statistical approaches as discriminating features, with IL-1a, IL-2 and IFN-γ also selected in one model or the other. This supported an assignment accuracy of better than 80% at a confidence level of 0.95 into PI-CFS versus recovered controls.

**Conclusion:**

These results suggest that co-expression patterns in as few as 5 cytokines associated with Th17 function may hold promise as a tool for the diagnosis of post-infectious CFS.

## Background

Chronic Fatigue Syndrome (CFS) is a complex and poorly understood illness that affects between 1 and 4 million individuals [1,2] and costs an estimated $35 billion per year in lost productivity and health care [3,4]. Persistent and unexplained fatigue has been observed as a sequela of acute infection since the first half of the 20th century and the period’s notable outbreaks of infectious disease (e.g., polio epidemics, influenza pandemics). From 1934 to 1984, there were several reports of infectious disease outbreaks simulating poliomyelitis [5,6] though no causal pathogen was isolated from the majority of the cases. Clinical investigation of individuals who did not recover several years after initial infection showed persistent symptoms of fatigue, sleep disturbance and cognitive impairment [7,8]. Following an outbreak investigation in 1984 by the Centers for Disease Control and Prevention (CDC) in Incline Village, Nevada [9], an international group of medical experts defined this protracted post-infection illness as chronic fatigue syndrome (CFS) [10]. Since then, post-infection illness that lasts for more than six months is a central component of CFS, and studies have shown that the incidence of CFS following infection with a number of different pathogens is about 10% [11-14]. The pathogens that most commonly lead to CFS in prospective studies are those that cause infectious mononucleosis (IM)/glandular fever (mainly Epstein-Barr virus (EBV); occasionally cytomegalovirus (CMV), human herpesvirus 6, hepatitis A, and adenovirus [11], Q fever and Ross River virus (mainly in Australia [13]), enteroviruses [15] and parvovirus B19 [16,17]. Indeed in recent work by Kerr and colleagues, antibody testing for EBV, enterovirus, Coxiella burnetii (causative agent of Q fever) and parvovirus B19 revealed evidence of subtype-specific relationships for EBV and enterovirus, two of the most common infectious triggers of CFS/ME [18].

There remains therefore an overwhelming body of evidence reinforcing the link to an infectious etiology in at least a subset of CFS patients, despite the fact that specific viral serology are not required for diagnosing CFS [19,20]. Specifically, there is a notable and long-standing association of CFS with EBV infection. In our previously published analyses of the adolescent cohort presented here [14,21-24], we reported a diagnosis of CFS at 6, 12 and 24 months following IM, in 13%, 7% and 4%, of the subjects respectively. Ninety percent of CFS subjects at 6 months were female as were all cases at 12 (n = 22) and 24 (n = 13) months following IM. This is consistent with the rates observed by Buchwald and colleagues (2000) [12] who found 12% of subjects had not recovered and were reporting symptoms of fatigue and impaired functioning 6 months following onset of IM, 76% of whom were female. Nearly identical results were also reported in an Australian cohort where 11% and 9% of subjects satisfied the case definition for CFS at 6 and 12 months following onset of IM, Q fever and Ross River fever (RRF) [13].

Investigators have sought to understand the underlying causes of these persistent symptoms by surveying immune signaling in the CFS patient population. Early measurements of cytokine concentrations in blood samples from this broader population produced widely discordant results due mainly to differences in case definition and laboratory protocols [25]. Limiting factors also include the focus on a varied and narrow selection of cytokines [26] as well as a conventional univariate analysis that does not account for the context-specific expression of cytokines [27]. Only one previous study has prospectively evaluated cytokine production in CFS patient populations where a uniform infectious trigger was supported by serology. Hickie et al. (2006) [13] measured the concentrations of 8 cytokines: IL-1b, IL-2, IL-4, IL-6, IL-10, IL-12, TNF-α, and IFN-γ in serum and culture supernatants of peripheral blood mononuclear cells collected 1, 2, 3, 6 and 12 months following the acute illness (IM, RRF or Q fever). The participants were 22 subjects with confirmed PI-CFS (11 EBV, 6 RRV, and 5 Q fever) and 42 control subjects (17 EBV, 14 RRV, and 11 Q fever) matched for age from the same cohort who had recovered within 6 weeks of symptom onset. The analyses were performed on all PI-CFS subjects as a single group, combining the 11 EBV patients with those infected by a virus uncommon outside Australia. They found no significant differences between PI-CFS cases and recovered controls in serum or culture supernatants [28]. However, serum cytokine levels were almost exclusively below the assay detection limit (8–15 pg/ml) and supernatants were cultured from cryopreserved PBMCs. As a result several unanswered questions remain. For example, do key CFS-specific cytokines remain unmeasured? Perhaps most importantly, could PI-CFS cytokine signatures reflect pathogen-specific (and gender-specific) variations in immune response? If so these may be lost when pooling subjects with different infectious triggers (and different genders).

In an attempt to address some of these questions, this secondary analysis has focused specifically on a group of female adolescents diagnosed PI-CFS as the result of a uniform and known pathogen, namely EBV. An extended survey of 16 cytokines was conducted in plasma using a modified chemiluminescent assay [27] and identified significant differences in IL-8 and IL-23 concentrations in the patient group at 24 months post-infection. Because cytokines are expressed in a coordinated fashion we also extended the analysis beyond the traditional univariate analysis to investigate combinatorial effects across multiple cytokines. Taken together, a classification of subjects based on levels of IL-2, 6, 8 and 23 supported assignment into the patient or control group with an accuracy exceeding 80% when applied relative to interferon gamma (IFN-γ) concentration. Interestingly the latter did not differ significantly in expression level across subject groups.

## Methods

### Cohort and clinical assessment

The cohort consisted of 301 adolescents recruited from the greater Chicago area upon diagnosis of monospot-positive acute IM, the presumption being that the vast majority of these cases were caused by EBV infection. Details of this cohort may be found in a recent report by Katz et al. (2009) [14]. In brief, adolescents were identified via school nurses (middle school, high school and college/university), pediatric practices, including the Pediatric Practice Research Group and the Virology Laboratory of Children’s Memorial Hospital. All prescribed treatments were recorded. Six months following their IM diagnosis, 286 (95%) subjects completed a telephone-based screening interview using the CFS Screening Questionnaire [29]. Based on the screening interview, 70 of these adolescents (24%) were assessed as not fully recovered. A clinical evaluation was completed on 53 (76%) of these 70 not fully recovered adolescents; 12 refused, 3 had exclusionary diagnoses and 2 did not meet study criteria. There was no significant difference in sex, family socioeconomic status or subject age between the group that completed the 6-month evaluation (n = 53), the group (n = 12) that refused and the group (n = 5) that was excluded (data not shown). Following the 6-month clinical evaluation, 39 of the 53 not fully recovered subjects who underwent clinical evaluation were classified as having CFS (13% of the original sample of 301 adolescents). Compared with the other 262 enrolled subjects in the cohort, 35 of the 39 subjects with CFS at 6 months were female (90%, vs 68%, p = 0.01 by Fisher’s exact test). There was no difference in race or socioeconomic status between the entire cohort and the subjects who went on to develop CFS (data not shown). Among the 14 other subjects completing the 6-month clinical evaluation, 1 had recovered between the time of the phone interview and the time he/she was seen in clinic and 13 were classified as CFS explained. There was no difference in family socioeconomic status or subject age between the group diagnosed with CFS (n = 39) and the group (n = 13) with CFS-explained (data not shown).

Thirty-six subjects diagnosed with CFS at the 6-month evaluation were re-evaluated at 12 months (3 were lost to follow-up): 11 recovered, 3 were now classified as CFS-explained, leaving 22 with a diagnosis of CFS (7% of the original sample; all females). At the 24-month evaluation, 3 more subjects with CFS were lost to follow-up since the 12-month evaluation. Of the 19 remaining subjects diagnosed with CFS at 12 months: 6 more recovered, 2 developed a reason for their CFS, 1 subject thought to have recovered at 12 months developed symptoms again and was reclassified as CFS, and 1 subject thought to have CFS-explained at 12 months now met criteria for CFS and had no predisposing reason, leaving 13 subjects (all females) who were classified as CFS 24 months following monospot-positive IM (4% of the original sample of 301 adolescents). Following the IM diagnosis, a telephone-screening interview also identified 50 recovered controls willing to come for a clinical evaluation. Adolescents who had not fully recovered and recovered controls underwent evaluation 6, 12 and 24 months post-IM. There was no difference in age, race or socioeconomic status between recovered adolescents who were and were not used as controls subjects (data not shown). At the 6-month evaluation recovered and non-recovered subjects were evaluated by 1 of 2 physicians experienced in diagnosing CFS (BZK or an adolescent specialist). At the 12 and 24-month visits, the subjects were evaluated in their home with the same blood and urine testing and the same history, interviews, and self-report measures used at six months, by a trained interviewer. All aspects of the study were approved by the Institutional Review Boards of Children’s Memorial Research Center and the College of Applied Sciences of the University of Illinois at Chicago. Secondary analysis of the biological samples and data was reviewed and approved by the Institutional Review Board of the University of Alberta. Diagnosis of CFS was based on the revision by Jason et al. (2006) [30] of the international CFS case definition [20], itself a revision of the of the Fukuda criteria [19], although all patients also met the international case definition of CFS [20]. When a well-recognized underlying condition such as primary depression could explain a subject’s symptoms then that subject was classified as having “CFS-explained”, and these subjects were not included in the analysis. Primary depression was defined based on the Structured Clinical Interview for the DSM-IV Child Version.

The clinical evaluation included laboratory tests to rule out medical causes of CFS (e.g., chemistry panel, complete blood count, erythrocyte sedimentation rate, liver chemistries, urine toxicology, urinalysis and thyroid function tests). The examining physician at 6 months made a diagnosis of CFS, CFS-explained, or recovered on each subject. These diagnoses were then blindly reviewed by an expert panel using the Jason revision for pediatrics [30] of the Fukuda criteria [19] before being permanently assigned to a subject. Each subject also completed the Chalder Fatigue Scale at 6, 12 and 24 months [31], along with the Youth Medical Questionnaire, Modifiable Activity Questionnaire, Sleep Assessment Questionnaire, and the Structured Clinical Interview for the DSM-IV Child Version, Child/Young Adult Behavior Checklist, Child/Young Adult Self-Report, and Life Events and Difficulty Questionnaire [30]. At 24-months post-IM onset, 13 subjects satisfied the criteria for CFS, all of whom were young women. Plasma samples were available for n = 9 of these 13 CFS patients 24-months after diagnosis with IM and 12 recovered controls, matched as closely as possible to the subjects with CFS by gender, age (+/- 1 year) and Tanner stage (4 or 5).

### Laboratory measurements

Morning fasting blood samples were collected into ethylene diamine tetra acetic acid (EDTA) anticoagulant tubes. Plasma was separated within 2 hours of collection and stored at -80^o^C until assayed. We measured 16 cytokines in plasma using Quansys reagents and instrument (Quansys Biosciences, Logan, Utah) in the same way as reported previously in a larger cohort of CFS subjects with unknown etiology and illness trigger [26]. The Quansys Imager, driven by an 8.4 megapixel Canon 20D digital SLR camera, supports 96 well plate based chemiluminescent imaging. The Q-Plex™ Human Cytokine - Screen (16-plex) is a quantitative enzyme-linked immunoabsorbent assay (ELISA)-based test where sixteen distinct capture antibodies have been absorbed to each well of a 96-well plate in a defined array. The range of the cytokine concentrations used in the standard calibration samples were adjusted for each cytokine along with sample exposure time to provide the most reliable comparison possible between CFS patients and controls across the range of cytokine concentrations known and expected in plasma. For the standard curves, we used the second order (k = 2) polynomial regression model (parabolic curve): Y_p_ = b_0_ + b_1_X_1_ + b_2_X_2_.. + b_k_X_k_, where Y_p_ is the predicted outcome value for the polynomial model with regression coefficients b_1_ to b_k_ for each degree and y intercept b_0_. The standard sample concentrations used to establish the calibration curves for each cytokine are listed in Additional file 1: Table S1; these confirm that the concentrations reported in study subjects are within the calibrated range for this assay.

### Statistical analyses

#### Comparative statistics

Each plasma sample was run in duplicate. To compare subject-to-subject variation with replicate error both the parametric n-way ANOVA and the nonparametric Kruskall-Wallis tests were used. Results presented in Additional file 1: Table S2 indicate that variations separating individuals was statistically greater than replicate error for most cytokines in at least one subject group or the other. The parametric n-way ANOVA applied to log-transformed values was generally more sensitive to these differences than the Kruskall-Wallis test. Replicate error in the measurement of IL-1a, IL-10 and IL-17 did not support subject-to-subject resolution within either group regardless of the statistical test used. These were nonetheless adequate for use in comparisons across patient groups as a whole.

To assess the significance of differences in the mean expression of individual cytokines separating subject groups we used a standard parametric t test after performing a logarithmic transformation. In conjunction with this a nonparametric Wilcoxon rank-sum test was used to compare the difference in group-wise median expression for each cytokine. Values below detection limit were replaced with the lowest concentration recorded for each specific cytokine within each subject group.

#### Classification analysis

As an extension of this group-wide analysis of expression levels, cytokines were ranked according to their classification of individual subjects based on their associated receiver operating characteristic (ROC) curves and on the U-statistic of a two-sample unpaired Wilcoxon ranksum test. In addition to an unadjusted ranking, a relative ranking was also computed that accounted for the cross-correlation (CC) existing between individual cytokines. In this adjustment correlation information was used to reweight the Z value of a candidate cytokine using Z_i_ (1-α ‾r _ij_), where ‾r_ij_ is the average of the absolute values of the cross-correlation coefficient between the candidate i and all previously selected cytokines j = 1 through i. The weighting factor α (alpha) is a scalar value between 0 and 1. According to this adjustment a large value of ‾r_ij_ outweighs the significance statistic Z making new candidates that are highly correlated with previously ranked cytokines less likely to be selected next.

The use of cytokines in combination with one another was also considered. For this, linear discriminant classification models were constructed iteratively using randomly selected subsets of cytokines and their performance evaluated. All cytokines were normalized to have a mean value of 0 and a variance of 1.0. Classifiers were evaluated based on their overall error rate (incorrectly classified samples / total classified samples) in assigning subjects to their proper diagnostic group. Only classifiers with overall error rate of less than 20% were accepted into the pool of candidates. In addition the posterior probability of the discriminant analysis was used to invalidate candidate class assignments with low confidence (< 0.90) and label any such instance as an inconclusive classification. Furthermore a strict linear classification model was selected to favor subsets of cytokines that were minimally redundant. Traditional ordinary least squares models will perform poorly when correlated or collinear terms are used together due to variance inflation [32]. Final correction of the linear classifier for collinearity in the selected cytokine subsets was performed using a diagonal covariance matrix estimate. According to this model an observed row x from the sample array is classified into group I rather than group J if 0 < B_0_ + x*B, where the coefficient vector B and intercept vector B_0_ are estimated from the data.

In order to assess the robustness of models constructed using the all-possible-subsets approach described above, an analysis of this data using a sequential stepwise variable selection [33] was also conducted. Using this algorithm, a linear classification model was constructed by selecting cytokines sequentially based on their respective partial-F test values. Cytokines with a p(partial F) < 0.05 were selected for recruitment into the regression model while those currently in the model but showing a p(partial F) > 0.10 were pruned. All calculations described above were performed using the *classify, classperf, rankfeatures, randfeatures and stepwisefit* functions available in the MatLab Statistics Toolbox and the MatLab Bioinformatics Toolbox (The MathWorks, Inc., Natick, MA).

## Results

### Cytokine expression in PI-CFS patients

**Figure 1:**
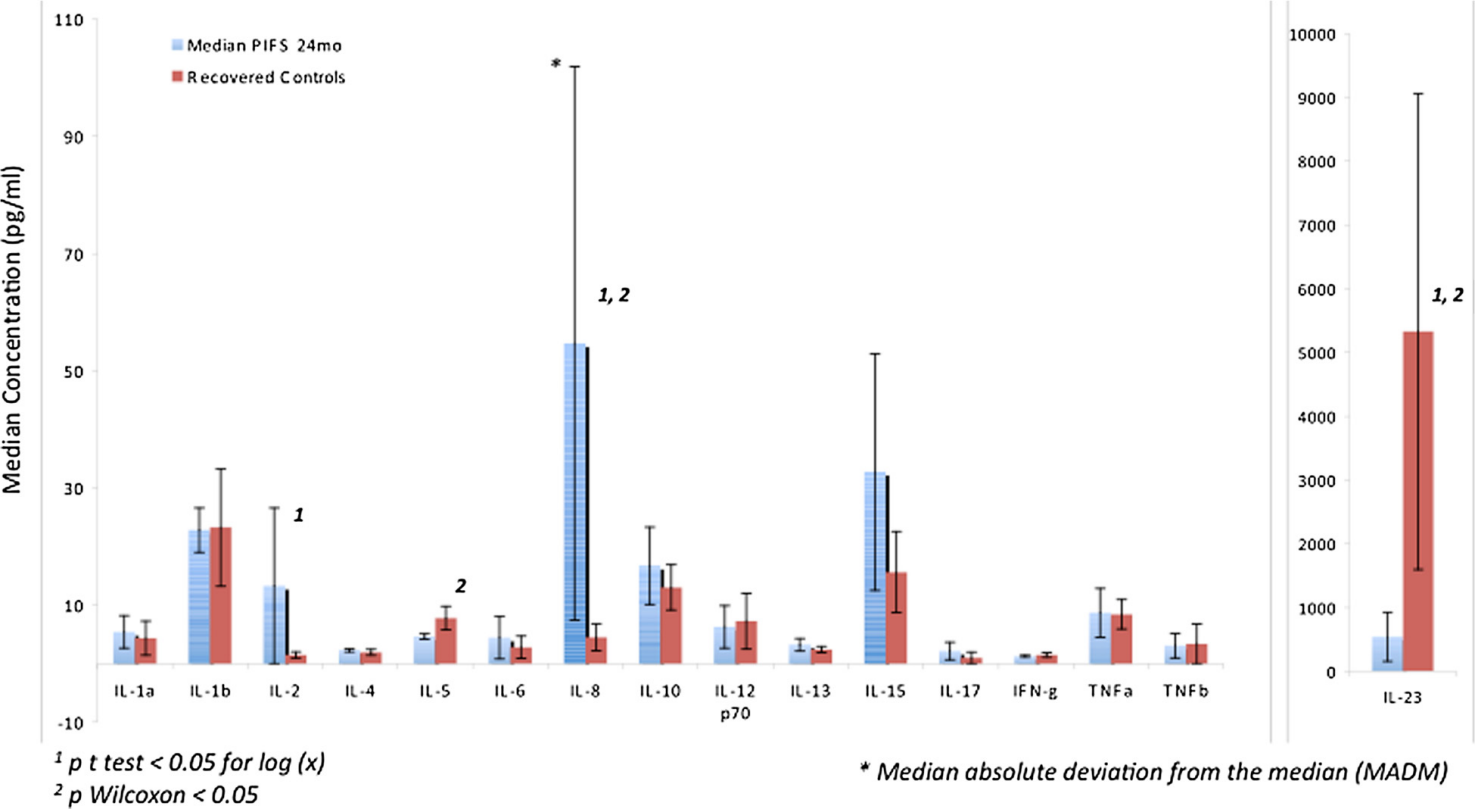
**Cytokine Expression in Post-mononucleosis Chronic Fatigue.** Cytokine Expression Median concentration (with median absolute deviation from median) of 16 cytokines measured in peripheral blood plasma from n = 12 adolescents recovering normally at 24 months from infectious mononucleosis (IM) and n = 9 patients suffering from post-infectious chronic fatigue syndrome (PI-CFS).

Summary statistics for group-wise expression of cytokines in PI-CFS patients and subjects who recovered normally from IM are presented graphically in Figure 1 (see also Additional file 1: Table S3). Briefly these show that IL-8 was significantly elevated in median expression (p ≤ 0.05) in PI-CFS while IL-23 was significantly decreased. We also found IL-2 expression to be elevated in PI-CFS though this reached statistical significance for the t-test only. Similarly reduced expression of IL-5 in PI-CFS was significant based on the Wilcoxon rank sum test only. A trend towards increased IL-13 expression was also observed (p ~ 0.07).

### Ranking individual cytokine markers

Univariate analysis indicated that cytokines IL-2, IL-5, IL-8 and IL-23 were significantly different, in either mean or median expression, in PI-CFS patients compared to subjects that had recovered. To investigate these differences we estimated how individual cytokines might rank on the basis of their performance in assigning subjects to the correct diagnostic class, namely PI-CFS or recovered controls. As expected, the results presented in Table 1 indicate that as individual biomarkers IL-5, IL-8 and IL-23 ranked consistently in the top 3 positions based on both the area under the receiver operating characteristic curve (AUC) and the Wilcoxon U statistic. In terms of AUC, IL-8 was the single best discriminator between groups (0.34 above 0.50). In comparison IL-5 and IL-23, the next best markers represented a 40% decrease in AUC (0.20 above 0.50). With respect to the U statistic IL-8 and 23 were virtually equivalent with the next best marker leading to a 20% decrease in reliability. The next 3 positions were occupied by a combination of IL-2, IL-13, and IL-15 as well as IFN-γ, TNF-α and TNF-β depending on the criterion used. However, these corresponded to a greater than 50% reduction in the AUC and a 40% decrease in the U statistic when compared to IL-8 and IL-5 respectively. This ranking was slightly different when we corrected for cross-correlation between cytokines by reweighting the Z statistic using an alpha value of α=1 as described above (2.3.2 of the Methods section). Once again IL-5, 8 and 23 were selected as the top ranking cytokines. The next 3 positions were occupied by a combination of the same cytokines as before with the exception that IL-15 was replaced by IL-6. Once again these corresponded to a loss in performance of greater than 50% in both AUC and U metrics when compared to IL-5 or IL-8.

**Table 1:** Individual Cytokines as Illness Markers

Ranked Features PI-CFS vs Ctrl**			**CC weight alpha=0**	
**ROC**		**AUC-0.50**	**Wilcoxon**	**U statistic**
IL-8	(pg/ml)	0.34	IL-5	0.31
IL-5	(pg/ml)	0.20	IL-23	0.30
IL-23	(pg/ml)	0.20	IL-8	0.24
IL-13	(pg/ml)	0.16	IFN-g	0.18
IL-2	(pg/ml)	0.16	TNFa	0.13
IL-15	(pg/ml)	0.14	TNFb	0.13
IL-17	(pg/ml)	0.13	IL-12p70	0.10
IL-6	(pg/ml)	0.10	IL-1b	0.07
IL-10	(pg/ml)	0.08	IL-1a	0.06
IFN-g	(pg/ml)	0.07	IL-13	0.06
IL-4	(pg/ml)	0.07	IL-2	0.06
TNFb	(pg/ml)	0.04	IL-15	0.04
IL-1a	(pg/ml)	0.04	IL-17	0.04
IL-1b	(pg/ml)	0.04	IL-4	0.03
TNFa	(pg/ml)	0.03	IL-10	0.03
IL-12p70	(pg/ml)	0.01	IL-6	0.00
Ranked Features PI-CFS vs Ctrl**			**CC weight alpha=1.0**	
**ROC**		**AUC-0.50**	**Wilcoxon**	**U statistic**
IL-8	(pg/ml)	0.34	IL-5	0.31
IL-23	(pg/ml)	0.20	IL-8	0.24
IL-5	(pg/ml)	0.20	IL-23	0.30
IL-2	(pg/ml)	0.16	TNFb	0.13
IL-13	(pg/ml)	0.16	IFN-g	0.18
IL-6	(pg/ml)	0.10	TNFa	0.13
IL-17	(pg/ml)	0.13	IL-1a	0.06
IL-15	(pg/ml)	0.14	IL-12p70	0.10
IFN-g	(pg/ml)	0.07	IL-2	0.06
IL-10	(pg/ml)	0.08	IL-1b	0.07
IL-4	(pg/ml)	0.07	IL-13	0.06
IL-1a	(pg/ml)	0.04	IL-17	0.04
TNFb	(pg/ml)	0.04	IL-15	0.04
IL-1b	(pg/ml)	0.04	IL-10	0.03
TNFa	(pg/ml)	0.03	IL-4	0.03
IL-12p70	(pg/ml)	0.01	IL-6	0.00

Relative rank of individual cytokines based on their classification performance as evaluated by the area (AUC) under the receiver operating characteristic (ROC) curve and the Wilcoxon U statistic. Values without cross-correlation adjustment (CC Weight α=0) and with full cross-correlation adjustment are shown (CC weight α=1).

** Note: values were not log transformed since ROC rank is non-parametric.

### A candidate cytokine-based signature for IM-induced chronic fatigue

**Figure 2:**
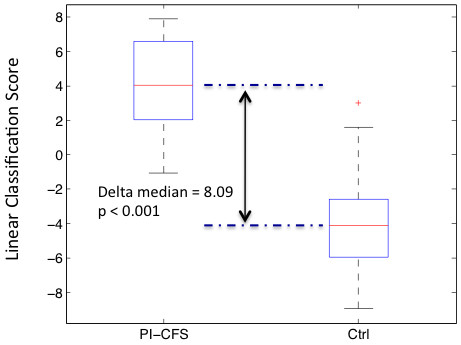
**A Cytokine-based Subject Classification.** Linear classification scores for the assignment of subjects to the overall PI-CFS group obtained using the simple linear classifier 0 > B_0_ + B*X where X is the normalized concentration of cytokines IL-2, 6, 8 23 and IFN-γ.

For all their merit individual cytokines are not without their limitations as biomarkers. Their use in combination has the potential to improve classification performance by introducing immune context. Cytokines are not expressed independently but instead their patterns arise as a result of one or several basic immune processes acting in concert. This limits the expression of cytokines to specific patterns and deviation from these patterns may be indicative of illness even though the cytokines in question may still be within expected ranges when considered individually. Not only is analysis of co-expression more representative of the underlying biology but from a purely statistical perspective, these measurements are subject to various levels of noise and the use of a weighted average score across several cytokines promises to be much more reliable. Indeed, in our analysis a minimal set of 5 cytokines was necessary to satisfy the criterion of better than 80% accuracy at a confidence level of 0.95 (Figure 2). The subset of 5 cytokines that maximized the performance of a simple linear classification model with minimal redundancy consisted of IL-2, IL-6, IL-8, IL-23 and IFN-γ. As shown in Table 2 this classification model supported a specificity of 88% at a sensitivity of 94% (PPV = 0.85, NPV = 0.95) when applied to the training data of adolescent PI-CFS patients and recovered controls. Only 7 of the 42 individual samples received an inconclusive assignment when a minimal confidence level of 0.95 was applied. In comparison classifiers constructed with cytokines best ranked on the basis of their individual performance produced much higher levels of uncertainty. For example the 5 cytokines ranked best in terms of contribution to the AUC, namely IL-8, IL-5, IL-2, IL-23 and IL-13, produced over 26 out of 42 inconclusive class assignments at a confidence threshold of 0.95. Adjusting further for the partial correlation between these cytokines (collinearity) only reduced this to 22 inconclusive class assignments. Similarly a classification based on IL-5, IL-23, IL-8, IFN-γ and TNF-β, ranked best on the basis of the U statistic, produced 29 inconclusive assignments in the set of 42 individual samples. Once again 24 of these remained after correction for correlation between cytokines (collinearity). The general performance characteristics for these alternate models are summarized in Table 2.

**Table 2:** **Performance Characteristics**^***1***^**of Subject Classification using Cytokine Combinations**

	**Optimal Linear model subset**			**Top ranking AUC with CC correction**			**Top ranking U statistic with CC correction**		
	**Classification based on IL-2, 6, 8, 23, IFNg**			**Classification based on IL-2, 5, 8, 13, 23**			**Classification based on IL-5, 23, 8, IFNg, TNFb**		
	**Linear cw 90% Conf**	**Linear uncorrected**		**Linear cw 90% Conf**	**Linear uncorrected**		**Linear cw 90% Conf**	**Linear uncorrected**	
Correct Rate	0.97	0.90		0.94	0.86		0.92	0.79	
Error Rate	0.03	0.10		0.06	0.14		0.08	0.21	
									
Inconclusive Rate	0.17	0.00		0.62	0.00		0.69	0.00	
Classified Rate	0.83	1.00		0.38	1.00		0.31	1.00	
									
Sensitivity	0.72	0.94		0.33	0.83		0.33	0.67	
Specificity	0.88	0.88		0.38	0.88		0.25	0.88	
Positive Predictive Value	0.81	0.85		0.29	0.83		0.25	0.80	
Negative Predictive Value	0.81	0.95		0.43	0.88		0.33	0.78	
									
Positive Likelihood	5.78	7.56		0.53	6.67		0.44	5.33	
Negative Likelihood	0.32	0.06		1.78	0.19		2.67	0.38	

Classification models based on random all-possible-subset selection of 5 cytokines as well as the top 5 ranking cytokines based on individual contribution to the AUC and the U statistic.

^*1*^ Correct Rate is defined as (correctly classified samples)/ (all *classified* samples); Error rate as (incorrectly classified samples)/ (all *classified* samples); Inconclusive Rate is defined as (non-classified samples) / (total number of samples); Classified rate is (classified samples) / (total number of samples); Sensitivity is defined as (correctly classified positives) / (true positives); Specificity is (correctly classified negatives) / (true negatives); Positive Predictive Value is (correctly classified positives) / (positive classified); Negative Predictive Value is (correctly classified negatives) / (negative classified); Positive Likelihood is Sensitivity / (1 – Specificity); Negative Likelihood is (1 – Sensitivity) / Specificity.

Even though IL-5 ranked highly in terms of individual performance it was not selected as a contributor to the classification model. To explore this further we examined the correlation of IL-5 with other cytokines. The cross-correlation patterns existing between cytokines in the PI-CFS patient set as well as in the recovered control group are presented in Additional file 1: Table S4. These indicate strong and significant (p < 0.05) correlations between IL-5 and IL-23 as well as with IFN-γ in the PI-CFS patient set (r = 0.65, 0.77; p < 0.01). Truncation of these terms and substitution with IL-5 into the random subset model was explored as a strategy for alleviating these redundancies. In all cases classification performance was significantly reduced (Additional file 1: Table S5). This suggests that IL-23 and IFN-γ are better suited than IL-5 to support class assignment based on IL-2, 8 and 6.

In order to test the robustness of this combination of cytokines, a stepwise selection algorithm was also applied to the same data. Stepwise selection produced a linear classification model that was once again based on a subset of 5 cytokines. In this sequential model-building scheme, IL-6, 8 and 23 were again chosen as significant markers of PI-CFS. Also selected were IL-1a and IL-13. Together theses 5 cytokines supported the assignment of samples to their correct diagnostic class with a specificity of 94% at a sensitivity of 91% (PPV = 0.95, NPV = 0.89) (Table 3). Using the consensus terms shared by both the all-possible-subsets (Table 2) and stepwise models leads to a classification model based on IL-6, 8 and 23 alone. This reduced consensus model supported a specificity of 88% at a sensitivity of 83% (PPV = 0.91, NPV = 0.80) (Table 3). Because samples from 4 PI-CFS patients were unavailable for analysis a set of simulations was used to test the stability of the above-mentioned models to inclusion of these subjects. In a worst-case scenario these patients were included in the analysis and assigned cytokine profiles selected randomly from the set of healthy normal profiles. With each random assignment a stepwise regression model was constructed. In 50 repeated assignments, IL-8 was unanimously selected in all regression models, while IL-6 ranked second with recruitment into 41 of 50 models (Additional file 1: Table S6). Of the initial classification model IL-6, 8 and 23 were selected by two different regression algorithms with simulations showing IL-6 and 8 to be particularly robust choices for this data.

**Table 3:** Stepwise and Consensus Classification Models

**Classification based on**	**Stepwise model of IL-1a, 6, 8,13, 23**	**Consensus model of IL-6, 8, and 23**	**Minimal model of IL-6 and 8**
Correct Rate	0.93	0.86	0.79
Error Rate	0.07	0.14	0.21
			
Inconclusive Rate	0.00	0.00	0.00
Classified Rate	1.00	1.00	1.00
			
Sensitivity	0.92	0.83	0.79
Specificity	0.94	0.89	0.78
Positive Predictive Value	0.96	0.91	0.83
Negative Predictive Value	0.89	0.80	0.74
			
Positive Likelihood	16.50	7.50	3.56
Negative Likelihood	0.09	0.19	0.27

Performance of linear classification models based on (i) a stepwise selection of IL-1a, 6, 8, 13 and 23, (ii) the intersecting subset of IL-6, 8 and 23 found in both stepwise and all-possible-subsets models and (iii) a core selection of IL-6 and 8, the two most robust choices based on simulations with random data substitutions.

## Discussion

In this work we studied a population of adolescents that developed CFS as a sequela of monospot-positive IM and compared them to control subjects who recovered normally from the same infection. Approximately 4% of subjects (or 13 individuals) diagnosed with IM fit the case definition for CFS at 24 months, a number consistent with other prospective studies of infectious onset CFS [12,13]. In comparing the concentrations of 16 cytokines between cases and recovered controls we found significantly different levels of IL-8, IL-23 and possibly IL-2 and IL-5 in plasma samples from CFS patients. Increased IL-8 and decreased IL-5 is a pattern also seen asthma [34] and in B cell chronic lymphocytic leukemia (B-CLL) [35]. Most striking were differences in IL-23, a cytokine expressed by dendritic cells and macrophages. Though not required for the generation of Th17 cells from naïve CD4+ T cells, IL-23 is nonetheless essential for the full and sustained differentiation of the Th17 cell subset [36]. Median concentration of this cytokine was significantly lower in patients with CFS than in recovered controls. IL-23 expression is highly inducible in PHA-stimulated CD4+ cells, in particular when primed with IFN-γ, suggesting a potential role for IL-23 in Th1 response [37]. We found decreased expression of IL-23 in CFS patients despite comparable levels of IFN-γ. In addition we found levels of IL-17 to be similar despite elevated levels of known antagonist IL-2 [38]. Consistent with this, our previous analysis of cytokine expression in a population of adult subjects with CFS of unknown etiology [27] indicated an abnormally subdued IL-23/Th17/IL-17 response to elevated levels of known inducers IL-1b and IL-6 [39].

The above-mentioned discrepancies emphasize the importance of immune context. Indeed it was necessary to use the combined expression of 5 cytokines to support a robust separation of patients from recovered controls. Ultimately the context linking the expression of these cytokines arises from one or several basic immune processes acting in concert. Here the choice of cytokines in both the all-possible-subsets and stepwise regression models is interesting in that the majority of these are either directly or indirectly related to Th17 response [39]. In this analysis IL-6, 8 and 23 were selected by two different statistical approaches, with IL-1a, IL-2 and IFN-γ also selected in one model or the other. It is interesting to note that neither IL-1a, nor IL-6 or IFN-γ were differentially expressed across groups even though they contributed significantly to the classification of these subjects. IL-1, 6 and 23 are known inducers of Th17 response, which in turn is a producer of IFN-γ [39]. Conversely IL-2 is generally known as a Th17 antagonist [38]. Liu et al. (2007) [40] describe a mechanism by which IL-1 induces the production of IL-23 via NF-kappa B activation, which in turn promotes the production of IL-6 and 8 in human fibroblast-like synoviocytes from rheumatoid arthritis patients. Though this is still poorly understood, recent evidence in animal models supports a role for EBV and EBV-inducible genes in the pathogenic modulation of Th17 response [41,42]. This may involve TLR9 which has been shown recently to play a significant role in the recognition of EBV by primary dendritic cells (DC), as indicated by a marked inhibitory effect on their synthesis of IFN-α, IL-6, and IL-8 [43]. Collectively the current analysis, as well as results from our previous work [27], suggests that illness-specific differences in the regulation of Th17 response may be a shared component in a significant subset of CFS cases.

When comparing this work with results from other studies it is important to remember the type of sample and the specific patient population studied. For example, recent work by Brenu et al. (2011) [44] demonstrated higher levels of IL-10, IFN-γ and TNF-α in CFS. However this was measured in mitogen-stimulated CD4+ cell cultures not in plasma. Similar in vitro protocols were used in earlier work by Amel Kashipaz et al. (2003) [45] and Skowera et al. (2004) [46]. Using the same sample type and laboratory protocol applied here, we recently reported higher levels of IL-5 and lower levels of IL-8 in CFS subjects compared to healthy controls, while levels of IL-23 showed little difference [27]. However both the work of Broderick et al. (2010) [27] and Brenu et al. (2011) [44] were conducted in a much broader CFS patient population where the illness trigger was not uniform. In addition the control population consisted of otherwise healthy individuals not individuals recovering from acute infection. Estimates of the number of patients with CFS who can date their illness to a specific (presumably infectious) acute illness range from 20- 90%, with the highest percentages being recorded in adolescent populations [8,47-53]. Accordingly our current results align closely with those of another study conducted specifically in a post-infectious patient population by Vollmer-Conna et al. (2007) [28]. The authors found no differences in the concentrations of IL-1b, IL-2, IL-4, IL-6, IL-10, IL-12, TNF-α, and IFN-γ analyzed in serum. With the possible exception of elevated IL-2, we also did not find differences in the levels of these cytokines. Levels of IL-5, 8 and 23 were not measured in this earlier study.

Limitations of our current study include the fact that it reports on a small cohort. This is important when considering the general applicability of our statistical classification model in particular. The hope is that this subset of subjects is somewhat representative of a larger PI-CFS patient population. CFS is well known for its heterogeneous presentation; however it can be argued that much of this is the result of varied etiology and unknown triggers. Our results are derived from a prospective study and constitute an important first look at the molecular phenotyping of persistent fatigue in a cohort with a uniform and known infectious trigger; namely infectious mononucleosis. While this trigger does not explain all presentations of CFS, it does represent a significant subset of patients, in particular in the pediatric population. Moreover though specific to the EBV pathogen, elements presented here may also be of relevance to other forms of CFS with an infectious etiology. Constraints such as these further emphasize the importance of prospective studies and the need for much larger initiatives of this type in this and related patient populations.

## Conclusion

In conclusion, the co-expression profile of 5 of 16 cytokines measured in the plasma of subjects recruited in a 2-year prospective study of mononucleosis-induced chronic fatigue syndrome readily discriminates cases from recovered control subjects and describes an atypical immune response in these individuals. In particular, irregular patterns of co-expression in IL-6, 8 and 23 were identified using two distinct statistical methodologies. These differences were especially evident when cast in the context of IL-1a, IL-2 and IFN-γ. This evidence points to a possible dysregulation of the innate system’s priming of the appropriate adaptive Th17 response in these subjects.

## Competing interests

The authors declare that they have no competing interests.

## Authors’ contributions

RT and BZK conducted the original study and all patient assessment, GB, BZK and SDV conceived the secondary analysis. MAF, NGK, BZK and MRGO performed the laboratory experiments. GB, HF and FAS performed all statistical analysis of the data. GB, BZK, MAF and SDV wrote the manuscript. All authors have read and approved the final manuscript.

## Supplementary Material

Additional file 1 **Table S1.** Assay performance measures for chemiluminescent determination of cytokine concentration. Described are the calibration standard sample concentrations used and the corresponding mean estimated concentration (pg/ml) based on duplicate measurements for each of the 16 cytokine species. Table S2. Significance of subject-to-subject changes in cytokine expression when compared with replicate error based on parametric a one-way ANOVA (ANOVA-1) and the non-parametric Kruskal-Wallis test. All concentrations were log-transformed. Table S3. Summary statistics (mean, standard deviation, median and median absolute deviation from the median - MADM) and statistical significance of the change in Chalder fatigue score and in the expression in 16 cytokines measured in plasma for the PI-CFS group compared to control subjects based on the standard two-tailed t-test and the non-parametric Wilcoxon test. Table S4. Pearson correlation coefficient *r* and associated null probability (p-value) for co-expression patterns existing between cytokines in the PI-CFS patient group and in the group of recovering control subjects. Table S5. Performance of alternative linear classification models were IL-5 has been substituted for IL-23 or IFN-γ or both to resolve collinearity issues with the latter by simple truncation. Statistics are shown for classification performance where a 90% confidence in assignment is imposed (Linear cw 90% conf.) and where the score alone is considered (Linear uncorrected). Table S61. Sensitivity study of n=4 absent PI-CFS profiles: in order to assess the impact of the n=4 sets of duplicate cytokine profiles missing from the n=13 PI-CFS subject group, a series of 20, 50 and 100 simulation experiments were conducted. In each experiment a random set of n=4 sets of duplicate samples were selected from the recovered control data and added to the PI-CFS set. A stepwise regression model was then used to select discriminating cytokines based on this artificially augmented data. Below is the frequency with which each cytokine was selected in the 50 regression models. Click here for file
