# Supplementary material for: Cytokine expression profiles of immune imbalance in post-mononucleosis chronic fatigue
Source: J Transl Med. 2012 Sep 13;10:191. doi: 10.1186/1479-5876-10-191 (PMC3480896; doi:10.1186/1479-5876-10-191)
Supplement: Additional file 1 — Table S1. Assay performance measures for chemiluminescent determination of cytokine concentration. Described are the calibration standard sample concentrations used and the corresponding mean estimated concentration (pg/ml) based on duplicate measurements for each of the 16 cytokine species. Table S2. Significance of subject-to-subject changes in cytokine expression when compared with replicate error based on parametric a one-way ANOVA (ANOVA-1) and the non-parametric Kruskal-Wallis test. All concentrations were log-transformed. Table S3. Summary statistics (mean, standard deviation, median and median absolute deviation from the median - MADM) and statistical significance of the change in Chalder fatigue score and in the expression in 16 cytokines measured in plasma for the PI-CFS group compared to control subjects based on the standard two-tailed t-test and the non-parametric Wilcoxon test. Table S4. Pearson correlation coefficient r and associated null probability (p-value) for co-expression patterns existing between cytokines in the PI-CFS patient group and in the group of recovering control subjects. Table S5. Performance of alternative linear classification models were IL-5 has been substituted for IL-23 or IFN-γ or both to resolve collinearity issues with the latter by simple truncation. Statistics are shown for classification performance where a 90% confidence in assignment is imposed (Linear cw 90% conf.) and where the score alone is considered (Linear uncorrected). Table S61. Sensitivity study of n=4 absent PI-CFS profiles: in order to assess the impact of the n=4 sets of duplicate cytokine profiles missing from the n=13 PI-CFS subject group, a series of 20, 50 and 100 simulation experiments were conducted. In each experiment a random set of n=4 sets of duplicate samples were selected from the recovered control data and added to the PI-CFS set. A stepwise regression model was then used to select discriminating cytokines based on this artificially augmented [file 1479-5876-10-191-S1.pdf]

**Table S1 (a)**. **Assay Performance Measures**. Calibration standard sample concentrations used and the corresponding mean estimated concentration (pg/ml) based on duplicate measurements for each cytokine.

| | **IL-1a** | | | **IL-1b** | |
|---|---|---|---|---|---|
| Dilution | Conc. (pg/ml) | Estimated Conc.(pg/ml) ** | | Conc. (pg/ml) | Estimated Conc.(pg/ml) |
| 1:1 | 6500.00 | 6590.30 | | 15000.00 | 14976.22 |
| 1:3 | 2166.67 | 2147.75 | | 5000.00 | 5063.16 |
| 1:9 | 722.22 | 737.18 | | 1666.67 | 1655.59 |
| 1:27 | 240.74 | 238.99 | | 555.56 | 569.90 |
| 1:81 | 80.25 | 74.37 | | 185.19 | 175.33 |
| 1:243 | 26.75 | 17.88 | | 61.73 | 51.34 |
| 1:729 | 8.92 | 20.74 | | 20.58 | 26.94 |
| | | | | | |
| | **IL-2** | | | **IL-4** | |
| Dilution | Conc. (pg/ml) | Estimated Conc.(pg/ml) | | Conc. (pg/ml) | Estimated Conc.(pg/ml) |
| 1:1 | 2700.00 | 2742.60 | | 2000.00 | 2010.02 |
| 1:3 | 900.00 | 921.40 | | 666.67 | 674.19 |
| 1:9 | 300.00 | 300.30 | | 222.22 | 218.46 |
| 1:27 | 100.00 | 99.86 | | 74.07 | 77.40 |
| 1:81 | 33.33 | 33.66 | | 24.69 | 24.02 |
| 1:243 | 11.11 | 10.54 | | 8.23 | 6.86 |
| 1:729 | 3.70 | 4.13 | | 2.74 | 2.06 |
| | | | | | |
| | **IL-5** | | | **IL-6** | |
| Dilution | Conc. (pg/ml) | Estimated Conc.(pg/ml) | | Conc. (pg/ml) | Estimated Conc.(pg/ml) |
| 1:1 | 1700.00 | 1716.81 | | 2500.00 | 2422.94 |
| 1:3 | 566.67 | 567.52 | | 833.33 | 835.02 |
| 1:9 | 188.89 | 189.14 | | 277.78 | 277.87 |
| 1:27 | 62.96 | 62.59 | | 92.59 | 92.36 |
| 1:81 | 20.99 | 21.45 | | 30.86 | 31.50 |
| 1:243 | 7.00 | 7.42 | | 10.29 | 9.61 |
| 1:729 | 2.33 | 1.20 | | 3.43 | 3.27 |
| | | | | | |
| | **IL-8** | | | **IL-10** | |
| Dilution | Conc. (pg/ml) | Estimated Conc.(pg/ml) | | Conc. (pg/ml) | Estimated Conc.(pg/ml) |
| 1:1 | 1000.00 | 1014.66 | | 1200.00 | 1195.97 |
| 1:3 | 333.33 | 340.79 | | 400.00 | 402.59 |
| 1:9 | 111.11 | 108.76 | | 133.33 | 130.72 |
| 1:27 | 37.04 | 38.75 | | 44.44 | 49.55 |
| 1:81 | 12.35 | 12.03 | | 14.81 | 8.52 |
| 1:243 | 4.12 | 3.48 | | 4.94 | 3.68 |
| 1:729 | 1.37 | 1.21 | | 1.65 | 3.42 |

** Average of 2 duplicate samples.

**Table S1 (b)**. **Assay Performance Measures (cont'd)**. Calibration standard sample concentrations used and the corresponding mean estimated concentration (pg/ml) based on duplicate measurements for each cytokine (cont'd).

| | IL-12 | | | IL-13 | |
|---|---|---|---|---|---|
| Dilution | Conc. (pg/ml) | Estimated Conc.(pg/ml) ** | | Conc. (pg/ml) | Estimated Conc.(pg/ml) |
| 1:1 | 5000.00 | 5135.38 | | 2500.00 | 2541.28 |
| 1:3 | 1666.67 | 1679.74 | | 833.33 | 834.09 |
| 1:9 | 555.56 | 551.65 | | 277.78 | 277.82 |
| 1:27 | 185.19 | 191.67 | | 92.59 | 92.79 |
| 1:81 | 61.73 | 57.04 | | 30.86 | 31.44 |
| 1:243 | 20.58 | 17.72 | | 10.29 | 8.78 |
| 1:729 | 6.86 | 8.00 | | 3.43 | 3.43 |
| | | | | | |
| | IL-15 | | | IL-17 | |
| Dilution | Conc. (pg/ml) | Estimated Conc.(pg/ml) | | Conc. (pg/ml) | Estimated Conc.(pg/ml) |
| 1:1 | 4000.00 | 4003.80 | | 2000.00 | 1978.70 |
| 1:3 | 1333.33 | 1340.39 | | 666.67 | 688.26 |
| 1:9 | 444.44 | 447.34 | | 222.22 | 223.15 |
| 1:27 | 148.15 | 144.33 | | 74.07 | 74.42 |
| 1:81 | 49.38 | 55.05 | | 24.69 | 23.15 |
| 1:243 | 16.46 | 21.86 | | 8.23 | 9.85 |
| 1:729 | 5.49 | 2.47 | | 2.74 | 0.62 |
| | | | | | |
| | IL-23 | | | IFNg | |
| Dilution | Conc. (pg/ml) | Estimated Conc.(pg/ml) | | Conc. (pg/ml) | Estimated Conc.(pg/ml) |
| 1:1 | 80000.00 | 80078.43 | | 600.00 | 599.75 |
| 1:3 | 26666.67 | 26658.14 | | 200.00 | 200.30 |
| 1:9 | 8888.89 | 8915.54 | | 66.67 | 66.48 |
| 1:27 | 2962.96 | 3036.90 | | 22.22 | 22.45 |
| 1:81 | 987.65 | 646.82 | | 7.41 | 7.25 |
| 1:243 | 329.22 | 39.37 | | 2.47 | 2.30 |
| 1:729 | 109.74 | 804.57 | | 0.82 | 0.74 |
| | | | | | |
| | TNFa | | | TNFb | |
| Dilution | Conc. (pg/ml) | Estimated Conc.(pg/ml) | | Conc. (pg/ml) | Estimated Conc.(pg/ml) |
| 1:1 | 2300.00 | 2343.72 | | 6000.00 | 6018.64 |
| 1:3 | 766.67 | 767.48 | | 2000.00 | 1991.79 |
| 1:9 | 255.56 | 255.89 | | 666.67 | 673.29 |
| 1:27 | 85.19 | 84.31 | | 222.22 | 219.33 |
| 1:81 | 28.40 | 29.71 | | 74.07 | 71.23 |
| 1:243 | 9.47 | 10.09 | | 24.69 | 20.96 |
| 1:729 | 3.16 | 1.79 | | 8.23 | 10.56 |

** Average of 2 duplicate samples.

**Table S2**. **Analysis of Variance**. Significance of subject-to-subject changes in cytokine expression when compared with replicate error based on parametric a one-way ANOVA (ANOVA-1) and the non-parametric Kruskal-Wallis test.

| | | p ANOVA-1 | | | p Kruskal-Wallis | |
|---|---|---|---|---|---|---|
| | | PI-CFS (Log) | Recovering Ctrl (Log) | | PI-CFS | Recovering Control |
| IL-1a | (pg/ml) | 0.305 | *0.123* | | 0.299 | 0.099 |
| IL-1b | (pg/ml) | 0.001 | 0.000 | | 0.100 | 0.040 |
| IL-2 | (pg/ml) | *0.064* | *0.015* | | 0.071 | 0.106 |
| IL-4 | (pg/ml) | 0.104 | 0.002 | | 0.173 | 0.235 |
| IL-5 | (pg/ml) | 0.000 | 0.041 | | 0.081 | 0.119 |
| IL-6 | (pg/ml) | 0.000 | 0.000 | | 0.036 | 0.038 |
| IL-8 | (pg/ml) | 0.000 | 0.000 | | 0.035 | 0.030 |
| IL-10 | (pg/ml) | 0.355 | 0.081 | | 0.381 | 0.069 |
| IL-12p70 | (pg/ml) | 0.023 | 0.079 | | 0.099 | 0.160 |
| IL-13 | (pg/ml) | 0.038 | 0.358 | | 0.105 | 0.356 |
| IL-15 | (pg/ml) | 0.000 | 0.000 | | 0.056 | 0.026 |
| IL-17 | (pg/ml) | *0.217* | *0.194* | | 0.226 | 0.378 |
| IL-23 | (pg/ml) | 0.000 | 0.000 | | 0.039 | 0.021 |
| IFN-g | (pg/ml) | 0.018 | 0.000 | | 0.145 | 0.047 |
| TNFa | (pg/ml) | 0.003 | 0.000 | | 0.135 | 0.103 |
| TNFb | (pg/ml) | *0.203* | *0.000* | | 0.181 | 0.312 |

**Table S3**. **Overview of Results**. Summary statistics and statistical significance of the change in Chalder fatigue score and in the expression in 16 cytokines measured in plasma for the PI-CFS group compared to control subjects.

| | | **PI-CFS** | | **Recovering Ctrl** | | **PI-CFS vs Ctrl** | |
|---|---|---|---|---|---|---|---|
| | | Mean (Std. Dev.) | Median (MADM) | Mean (Std. Dev.) | Median (MADM) | t test | Wilcoxon |
| Chalder Fatigue Score | | 18.33 (5.04) | 17.00 (3.00) | 11.00 (3.66) | 11.00 (0.50) | *0.000* | *0.000* |
| | | | | | | ** t test Log(x)* | |
| IL-1a | (pg/ml) | 23.04 (59.95) | 5.43 (2.76) | 5.82 (4.23) | 4.36 (2.91) | 0.439 | 0.657 |
| IL-1b | (pg/ml) | 31.11 (22.03) | 22.76 (3.80) | 50.56 (64.31) | 23.25 (9.99) | 0.788 | 0.684 |
| *IL-2* | (pg/ml) | 26.22 (29.65) | 13.29 (13.29) | 5.98 (10.55) | 1.50 (0.53) | *0.002* | 0.073 |
| IL-4 | (pg/ml) | 2.16 (0.45) | 2.31 (0.28) | 2.57 (2.16) | 1.96 (0.52) | 0.921 | 0.423 |
| *IL-5* | (pg/ml) | 6.90 (4.83) | 4.66 (0.46) | 7.88 (2.79) | 7.80 (2.03) | 0.110 | *0.026* |
| IL-6 | (pg/ml) | 34.51 (45.05) | 4.46 (3.63) | 10.72 (15.91) | 2.81 (1.93) | 0.153 | 0.280 |
| *IL-8* | (pg/ml) | 177.09 (173.55) | 54.64 (47.21) | 37.61 (92.38) | 4.52 (2.32) | *0.000* | *0.000* |
| IL-10 | (pg/ml) | 23.67 (17.69) | 16.70 (6.59) | 19.03 (17.07) | 13.04 (3.85) | 0.330 | 0.395 |
| IL-12p70 | (pg/ml) | 18.91 (29.46) | 6.29 (3.65) | 16.54 (21.24) | 7.27 (4.76) | 0.938 | 0.949 |
| IL-13 | (pg/ml) | 3.94 (2.70) | 3.23 (1.05) | 2.65 (1.24) | 2.43 (0.54) | 0.073 | 0.071 |
| IL-15 | (pg/ml) | 34.92 (24.00) | 32.72 (20.17) | 23.23 (17.88) | 15.64 (6.89) | 0.096 | 0.118 |
| IL-17 | (pg/ml) | 3.93 (5.21) | 2.16 (1.49) | 1.73 (2.03) | 1.00 (1.00) | 0.455 | 0.121 |
| *IL-23* | (pg/ml) | 19798.00 (33373.00) | 542.12 (385.45) | 14077.00 (13724.00) | 5329.60 (3731.60) | *0.012* | *0.030* |
| IFN-g | (pg/ml) | 1.60 (0.83) | 1.28 (0.16) | 2.25 (2.18) | 1.51 (0.38) | 0.338 | 0.423 |
| TNFa | (pg/ml) | 16.99 (21.15) | 8.70 (4.18) | 15.10 (15.40) | 8.45 (2.52) | 0.923 | 0.770 |
| TNFb | (pg/ml) | 3.50 (2.90) | 3.09 (2.11) | 11.42 (25.88) | 3.39 (3.39) | 0.335 | 0.769 |

**Table S4**. **Cytokine-cytokine Correlation Structure.** Pearson correlation coefficient r and associated null probability (p-value) for co-expression patterns existing between cytokines in the PI-CFS patient group and in the group of recovering control subjects.

**Correlation coefficients with p-value for log transformed concnetrations in all PI-CFS groups**

| | IL-1a | IL-1b | IL-2 | IL-4 | IL-5 | IL-6 | IL-8 | IL-10 | IL-12p70 | IL-13 | IL-15 | IL-17 | IL-23 | IFN-g | TNFa | TNFb |
|---|---|---|---|---|---|---|---|---|---|---|---|---|---|---|---|---|
| IL-1a | 1.00 (0.00) | 0.45 (0.06) | 0.17 (0.51) | 0.40 (0.10) | 0.43 (0.07) | -0.16 (0.51) | -0.27 (0.28) | 0.27 (0.28) | 0.54 (0.02) | 0.50 (0.04) | 0.34 (0.17) | 0.23 (0.36) | 0.34 (0.17) | 0.10 (0.68) | 0.28 (0.27) | 0.30 (0.23) |
| IL-1b | 0.45 (0.06) | 1.00 (0.00) | 0.62 (0.01) | 0.55 (0.02) | 0.65 (0.00) | -0.05 (0.85) | -0.23 (0.35) | 0.44 (0.07) | 0.61 (0.01) | 0.48 (0.04) | 0.52 (0.03) | 0.53 (0.02) | 0.30 (0.22) | 0.21 (0.41) | 0.54 (0.02) | 0.65 (0.00) |
| IL-2 | 0.17 (0.51) | 0.62 (0.01) | 1.00 (0.00) | 0.58 (0.01) | 0.37 (0.13) | 0.46 (0.05) | 0.36 (0.15) | 0.43 (0.08) | 0.54 (0.02) | 0.26 (0.30) | 0.82 (0.00) | 0.58 (0.01) | 0.23 (0.35) | 0.11 (0.67) | 0.34 (0.16) | 0.71 (0.00) |
| IL-4 | 0.40 (0.10) | 0.55 (0.02) | 0.58 (0.01) | 1.00 (0.00) | 0.20 (0.43) | -0.04 (0.86) | -0.17 (0.50) | 0.19 (0.45) | 0.67 (0.00) | 0.32 (0.20) | 0.40 (0.10) | 0.35 (0.15) | 0.17 (0.50) | -0.19 (0.45) | 0.22 (0.39) | 0.82 (0.00) |
| IL-5 | 0.43 (0.07) | 0.65 (0.00) | 0.37 (0.13) | 0.20 (0.43) | 1.00 (0.00) | -0.36 (0.14) | -0.43 (0.07) | 0.67 (0.00) | 0.63 (0.00) | 0.65 (0.00) | 0.39 (0.11) | 0.48 (0.05) | 0.77 (0.00) | 0.64 (0.00) | 0.70 (0.00) | 0.35 (0.16) |
| IL-6 | -0.16 (0.51) | -0.05 (0.85) | 0.46 (0.05) | -0.04 (0.86) | -0.36 (0.14) | 1.00 (0.00) | 0.93 (0.00) | -0.17 (0.50) | -0.19 (0.46) | -0.07 (0.79) | 0.63 (0.01) | 0.02 (0.94) | -0.39 (0.11) | -0.01 (0.96) | -0.17 (0.50) | 0.14 (0.59) |
| IL-8 | -0.27 (0.28) | -0.23 (0.35) | 0.36 (0.15) | -0.17 (0.50) | -0.43 (0.07) | 0.93 (0.00) | 1.00 (0.00) | -0.21 (0.41) | -0.27 (0.27) | -0.18 (0.48) | 0.51 (0.03) | 0.02 (0.94) | -0.31 (0.21) | 0.02 (0.94) | -0.21 (0.39) | 0.05 (0.83) |
| IL-10 | 0.27 (0.28) | 0.44 (0.07) | 0.43 (0.08) | 0.19 (0.45) | 0.67 (0.00) | -0.17 (0.50) | -0.21 (0.41) | 1.00 (0.00) | 0.38 (0.12) | 0.32 (0.20) | 0.25 (0.31) | 0.23 (0.35) | 0.57 (0.01) | 0.41 (0.09) | 0.62 (0.01) | 0.43 (0.07) |
| IL-12p70 | 0.54 (0.02) | 0.61 (0.01) | 0.54 (0.02) | 0.67 (0.00) | 0.63 (0.00) | -0.19 (0.46) | -0.27 (0.27) | 0.38 (0.12) | 1.00 (0.00) | 0.64 (0.00) | 0.53 (0.02) | 0.61 (0.01) | 0.68 (0.00) | 0.34 (0.17) | 0.66 (0.00) | 0.60 (0.01) |
| IL-13 | 0.50 (0.04) | 0.48 (0.04) | 0.26 (0.30) | 0.32 (0.20) | 0.65 (0.00) | -0.07 (0.79) | -0.18 (0.48) | 0.32 (0.20) | 0.64 (0.00) | 1.00 (0.00) | 0.50 (0.03) | 0.32 (0.20) | 0.58 (0.01) | 0.57 (0.01) | 0.44 (0.07) | 0.32 (0.20) |
| IL-15 | 0.34 (0.17) | 0.52 (0.03) | 0.82 (0.00) | 0.40 (0.10) | 0.39 (0.11) | 0.63 (0.01) | 0.51 (0.03) | 0.25 (0.31) | 0.53 (0.02) | 0.50 (0.03) | 1.00 (0.00) | 0.46 (0.05) | 0.26 (0.29) | 0.38 (0.12) | 0.38 (0.12) | 0.59 (0.01) |
| IL-17 | 0.23 (0.36) | 0.53 (0.02) | 0.58 (0.01) | 0.35 (0.15) | 0.48 (0.05) | 0.02 (0.94) | 0.02 (0.94) | 0.23 (0.35) | 0.61 (0.01) | 0.32 (0.20) | 0.46 (0.05) | 1.00 (0.00) | 0.43 (0.07) | 0.18 (0.47) | 0.53 (0.02) | 0.36 (0.14) |
| IL-23 | 0.34 (0.17) | 0.30 (0.22) | 0.23 (0.35) | 0.17 (0.50) | 0.77 (0.00) | -0.39 (0.11) | -0.31 (0.21) | 0.57 (0.01) | 0.68 (0.00) | 0.58 (0.01) | 0.26 (0.29) | 0.43 (0.07) | 1.00 (0.00) | 0.71 (0.00) | 0.61 (0.01) | 0.27 (0.27) |
| IFN-g | 0.10 (0.68) | 0.21 (0.41) | 0.11 (0.67) | -0.19 (0.45) | 0.64 (0.00) | -0.01 (0.96) | 0.02 (0.94) | 0.41 (0.09) | 0.34 (0.17) | 0.57 (0.01) | 0.38 (0.12) | 0.18 (0.47) | 0.71 (0.00) | 1.00 (0.00) | 0.63 (0.00) | 0.12 (0.63) |
| TNFa | 0.28 (0.27) | 0.54 (0.02) | 0.34 (0.16) | 0.22 (0.39) | 0.70 (0.00) | -0.17 (0.50) | -0.21 (0.39) | 0.62 (0.01) | 0.66 (0.00) | 0.44 (0.07) | 0.38 (0.12) | 0.53 (0.02) | 0.61 (0.01) | 0.63 (0.00) | 1.00 (0.00) | 0.46 (0.06) |
| TNFb | 0.30 (0.23) | 0.65 (0.00) | 0.71 (0.00) | 0.82 (0.00) | 0.35 (0.16) | 0.14 (0.59) | 0.05 (0.83) | 0.43 (0.07) | 0.60 (0.01) | 0.32 (0.20) | 0.59 (0.01) | 0.36 (0.14) | 0.27 (0.27) | 0.12 (0.63) | 0.46 (0.06) | 1.00 (0.00) |

**Correlation coefficients with p-value for log transformed concnetrations in Ctrl group only**

| | IL-1a | IL-1b | IL-2 | IL-4 | IL-5 | IL-6 | IL-8 | IL-10 | IL-12p70 | IL-13 | IL-15 | IL-17 | IL-23 | IFN-g | TNFa | TNFb |
|---|---|---|---|---|---|---|---|---|---|---|---|---|---|---|---|---|
| IL-1a | 1.00 (0.00) | 0.25 (0.24) | 0.21 (0.33) | 0.23 (0.28) | 0.12 (0.59) | -0.17 (0.42) | -0.50 (0.01) | 0.02 (0.94) | 0.32 (0.13) | 0.18 (0.41) | 0.12 (0.59) | 0.12 (0.57) | 0.00 (1.00) | 0.20 (0.36) | -0.04 (0.87) | 0.14 (0.52) |
| IL-1b | 0.25 (0.24) | 1.00 (0.00) | 0.33 (0.12) | 0.79 (0.00) | 0.62 (0.00) | 0.48 (0.02) | 0.01 (0.98) | 0.24 (0.26) | 0.65 (0.00) | 0.57 (0.00) | 0.90 (0.00) | 0.45 (0.03) | 0.22 (0.30) | 0.53 (0.01) | 0.83 (0.00) | 0.56 (0.00) |
| IL-2 | 0.21 (0.33) | 0.33 (0.12) | 1.00 (0.00) | 0.03 (0.89) | -0.04 (0.87) | 0.06 (0.77) | -0.05 (0.83) | 0.31 (0.14) | 0.37 (0.07) | 0.50 (0.01) | 0.32 (0.13) | 0.30 (0.15) | 0.44 (0.03) | -0.32 (0.12) | -0.01 (0.98) | 0.20 (0.35) |
| IL-4 | 0.23 (0.28) | 0.79 (0.00) | 0.03 (0.89) | 1.00 (0.00) | 0.31 (0.14) | 0.40 (0.06) | -0.05 (0.83) | -0.21 (0.32) | 0.64 (0.00) | 0.40 (0.05) | 0.57 (0.00) | 0.36 (0.08) | -0.03 (0.90) | 0.58 (0.00) | 0.77 (0.00) | 0.80 (0.00) |
| IL-5 | 0.12 (0.59) | 0.62 (0.00) | -0.04 (0.87) | 0.31 (0.14) | 1.00 (0.00) | 0.21 (0.33) | 0.04 (0.87) | 0.45 (0.03) | 0.42 (0.04) | 0.07 (0.76) | 0.77 (0.00) | 0.08 (0.72) | 0.20 (0.35) | 0.54 (0.01) | 0.54 (0.01) | -0.11 (0.61) |
| IL-6 | -0.17 (0.42) | 0.48 (0.02) | 0.06 (0.77) | 0.40 (0.06) | 0.21 (0.33) | 1.00 (0.00) | 0.67 (0.00) | 0.36 (0.09) | 0.29 (0.17) | 0.32 (0.12) | 0.44 (0.03) | 0.44 (0.03) | -0.11 (0.63) | 0.33 (0.11) | 0.46 (0.03) | 0.32 (0.13) |
| IL-8 | -0.50 (0.01) | 0.01 (0.98) | -0.05 (0.83) | -0.05 (0.83) | 0.04 (0.87) | 0.67 (0.00) | 1.00 (0.00) | 0.43 (0.04) | -0.15 (0.49) | -0.08 (0.71) | 0.09 (0.66) | 0.19 (0.38) | -0.19 (0.38) | -0.13 (0.56) | 0.09 (0.68) | -0.08 (0.71) |
| IL-10 | 0.02 (0.94) | 0.24 (0.26) | 0.31 (0.14) | -0.21 (0.32) | 0.45 (0.03) | 0.36 (0.09) | 0.43 (0.04) | 1.00 (0.00) | 0.05 (0.81) | 0.07 (0.73) | 0.45 (0.03) | 0.07 (0.76) | 0.41 (0.04) | -0.09 (0.68) | 0.15 (0.48) | -0.35 (0.09) |
| IL-12p70 | 0.32 (0.13) | 0.65 (0.00) | 0.37 (0.07) | 0.64 (0.00) | 0.42 (0.04) | 0.29 (0.17) | -0.15 (0.49) | 0.05 (0.81) | 1.00 (0.00) | 0.37 (0.08) | 0.57 (0.00) | 0.31 (0.14) | 0.36 (0.09) | 0.56 (0.00) | 0.53 (0.01) | 0.47 (0.02) |
| IL-13 | 0.18 (0.41) | 0.57 (0.00) | 0.50 (0.01) | 0.40 (0.05) | 0.07 (0.76) | 0.32 (0.12) | -0.08 (0.71) | 0.07 (0.73) | 0.37 (0.08) | 1.00 (0.00) | 0.51 (0.01) | 0.58 (0.00) | 0.19 (0.38) | 0.05 (0.83) | 0.35 (0.09) | 0.50 (0.01) |
| IL-15 | 0.12 (0.59) | 0.90 (0.00) | 0.32 (0.13) | 0.57 (0.00) | 0.77 (0.00) | 0.44 (0.03) | 0.09 (0.66) | 0.45 (0.03) | 0.57 (0.00) | 0.51 (0.01) | 1.00 (0.00) | 0.39 (0.06) | 0.39 (0.06) | 0.45 (0.03) | 0.81 (0.00) | 0.29 (0.17) |
| IL-17 | 0.12 (0.57) | 0.45 (0.03) | 0.30 (0.15) | 0.36 (0.08) | 0.08 (0.72) | 0.44 (0.03) | 0.19 (0.38) | 0.07 (0.76) | 0.31 (0.14) | 0.58 (0.00) | 0.39 (0.06) | 1.00 (0.00) | 0.13 (0.56) | 0.15 (0.49) | 0.33 (0.12) | 0.33 (0.11) |
| IL-23 | 0.00 (1.00) | 0.22 (0.30) | 0.44 (0.03) | -0.03 (0.90) | 0.20 (0.35) | -0.11 (0.63) | -0.19 (0.38) | 0.41 (0.04) | 0.36 (0.09) | 0.19 (0.38) | 0.39 (0.06) | 0.13 (0.56) | 1.00 (0.00) | 0.09 (0.67) | 0.26 (0.21) | -0.06 (0.79) |
| IFN-g | 0.20 (0.36) | 0.53 (0.01) | -0.32 (0.12) | 0.58 (0.00) | 0.54 (0.01) | 0.33 (0.11) | -0.13 (0.56) | -0.09 (0.68) | 0.56 (0.00) | 0.05 (0.83) | 0.45 (0.03) | 0.15 (0.49) | 0.09 (0.67) | 1.00 (0.00) | 0.57 (0.00) | 0.27 (0.20) |
| TNFa | -0.04 (0.87) | 0.83 (0.00) | -0.01 (0.98) | 0.77 (0.00) | 0.54 (0.01) | 0.46 (0.03) | 0.09 (0.68) | 0.15 (0.48) | 0.53 (0.01) | 0.35 (0.09) | 0.81 (0.00) | 0.33 (0.12) | 0.26 (0.21) | 0.57 (0.00) | 1.00 (0.00) | 0.48 (0.02) |
| TNFb | 0.14 (0.52) | 0.56 (0.00) | 0.20 (0.35) | 0.80 (0.00) | -0.11 (0.61) | 0.32 (0.13) | -0.08 (0.71) | -0.35 (0.09) | 0.47 (0.02) | 0.50 (0.01) | 0.29 (0.17) | 0.33 (0.11) | -0.06 (0.79) | 0.27 (0.20) | 0.48 (0.02) | 1.00 (0.00) |

**Table S5**. **Alternative Classification Models**. Performance of linear classification models were IL-5 has been substituted for IL-23 or IFN-γ or both to resolve collinearity issues with the latter by simple truncation. Statistics are shown for classification performance where a 90% confidence in assignment is imposed (Linear cw 90% conf.) and where the score alone is considered (Linear uncorrected).

| | Classification based on IL-2, 6, 8, 5, IFNg | | Classification based on IL-2, 6, 8, 23, 5 | | Classification based on IL-2, 6, 8, 5 | |
|---|---|---|---|---|---|---|
| | Linear cw 90% Conf | Linear uncorrected | Linear cw 90% Conf | Linear uncorrected | Linear cw 90% Conf | Linear uncorrected |
| Correct Rate | 0.96 | 0.88 | 0.92 | 0.86 | 0.96 | 0.83 |
| Error Rate | 0.04 | 0.12 | 0.08 | 0.14 | 0.04 | 0.17 |
| | | | | | | |
| Inconclusive Rate | 0.36 | 0.00 | 0.40 | 0.00 | 0.38 | 0.00 |
| Classified Rate | 0.64 | 1.00 | 0.60 | 1.00 | 0.62 | 1.00 |
| | | | | | | |
| Sensitivity | 0.61 | 0.89 | 0.56 | 0.89 | 0.56 | 0.89 |
| Specificity | 0.63 | 0.88 | 0.54 | 0.83 | 0.63 | 0.79 |
| Positive Predictive Value | 0.55 | 0.84 | 0.48 | 0.80 | 0.53 | 0.76 |
| Negative Predictive Value | 0.68 | 0.91 | 0.62 | 0.91 | 0.65 | 0.90 |
| | | | | | | |
| Positive Likelihood | 1.63 | 7.11 | 1.21 | 5.33 | 1.48 | 4.27 |
| Negative Likelihood | 0.62 | 0.13 | 0.82 | 0.13 | 0.71 | 0.14 |

**Table S6**. **Sensitivity study of n=4 Absent PI-CFS Profiles**. In order to assess the impact of the n=4 sets of duplicate cytokine profiles missing from the n=13 PI-CFS subject group, a series of 20, 50 and 100 simulation experiments were conducted. In each experiment a random set of n=4 sets of duplicate samples were selected from the recovered control data and added to the PI-CFS set. A stepwise regression model was then used to select discriminating cytokines based on this artificially augmented data. Below is the frequency with which each cytokine was selected in the 50 regression models.

| | **IL-1a** | **IL-1b** | **IL-2** | **IL-4** | **IL-5** | **IL-6** | **IL-8** | **IL-10** | **IL-12p70** | **IL-13** | **IL-15** | **IL-17** | **IL-23** | **IFNg** | **TNFa** | **TNFb** |
|---|---|---|---|---|---|---|---|---|---|---|---|---|---|---|---|---|
| 20 iterations | 13 | 0 | 0 | 1 | 0 | 17 | 20 | 0 | 0 | 7 | 1 | 2 | 8 | 0 | 0 | 0 |
| 20 iterations | 11 | 0 | 0 | 1 | 0 | 15 | 20 | 0 | 1 | 5 | 0 | 3 | 6 | 0 | 0 | 1 |
| 50 iterations | 34 | 1 | 0 | 0 | 0 | 42 | 50 | 0 | 2 | 15 | 1 | 4 | 17 | 1 | 0 | 2 |
| 50 iterations | 30 | 1 | 0 | 0 | 0 | 35 | 50 | 0 | 2 | 10 | 2 | 4 | 11 | 1 | 0 | 3 |
| 100 iterations | 70 | 7 | 0 | 1 | 0 | 77 | 99 | 1 | 3 | 28 | 7 | 12 | 29 | 1 | 0 | 2 |
| 100 iterations | 65 | 3 | 0 | 0 | 0 | 80 | 100 | 0 | 3 | 31 | 10 | 11 | 34 | 2 | 0 | 5 |
| | | | | | | | | | | | | | | | | |
| Average Selection Frequency (%) | 64 | 2 | 0 | 2 | 0 | 79 | 100 | 0 | 3 | 28 | 5 | 11 | 32 | 1 | 0 | 4 |
